# The Usefulness of Defining Rapid Virological Response by a Very Sensitive Assay (TMA) during Treatment of HCV Genotype 2/3 Infection

**DOI:** 10.1371/journal.pone.0120866

**Published:** 2015-08-28

**Authors:** Olav Dalgard, Michelle Martinot-Peignoux, Hans Verbaan, Kristian Bjøro, Helmer Ring-Larsen, Patrick Marcellin

**Affiliations:** 1 Department of Infectious Diseases, Akershus University Hospital, Lørenskog, Norway; 2 Faculty of Medicine, University of Oslo, Oslo, Norway; 3 Institut National de la Santé et de la Recherche Médicale, U-773, Centre de Recherche Biomédicale Bichat-Beaujon CRB3, Université Paris VII, Paris, France; 4 Medical Department, Malmø University Hopsital, Malmø, Sweden; 5 Clinic for specialized Medicine and Surgery, Oslo University Hospital, Rikshospitalet, Oslo, Norway; 6 Department of Pharmacology and Pharmacotherapy, University of Copenhagen, Copenhagen, Denmark; 7 Service d'Hépatologie, Hŏpital Beaujon, Clichy, France; Kaohsiung Medical University Hospital, Kaohsiung Medical University, TAIWAN

## Abstract

The aim of this study was to determine in patients with HCV genotype 2 or 3 the performance at week 4 of two assays with different sensitivities for HCV RNA detection, for the prediction of SVR and stratification for treatment duration (14 and 24 weeks). Recruitment was from two trials comparing 14 and 24 weeks treatment to patients with rapid virological response (RVR) (n = 550). RVR was originally defined as HCV RNA <50 IU/ml at week 4. Patients with an available frozen plasma sample drawn at week 4 and with follow-up data week 24 post-treatment were included (n = 429). HCV-RNA was prospectively measured with COBAS Amplicor V2, Roche (CA) (lower detection limit 50 IU/ml) and retrospectively assessed with VERSANT HCV-RNA Qualitative Assay, Siemens (TMA) (lower limit detection 10 IU/ml). Genotype 3 was present in 80% and genotype 2 in 20%. A SVR was achieved in 82%. At week 4 HCV-RNA was undetectable in 74.8% and 63% of serum samples tested with CA and TMA, respectively. CA undetectable/TMA positive was observed in 61/341 (18%) of the samples. In genotype 3 patients a relapse was seen in 9% of the patients with both CA and TMA undetectable and in 25% of the patients who were CA undetectable/TMA positive (p = 0.006). In patients allocated to 14 weeks treatment a relapse was observed in 11% of TMA undetectable patients and 26% of TMA positive (p = 0.031). In genotype 2 patients treated for 14 weeks relapse was observed in 6% of the patients with both CA and TMA undetectable week 4. Assays with high sensitivity for HCV RNA identifies patients at week 4 with high risk of virological relapse. We recommend that patients with genotype 3 and detectable HCV RNA at levels below 50 IU/ml do not receive truncated therapy with pegIFN and ribavirin.

## Introduction

Most patients with chronic hepatitis C virus (HCV) genotype 2 or 3 infection are currently treated with pegylated interferon α (pegIFN) and ribavirin for 24 weeks and 80% achieve a sustained virological response (SVR) to this treatment [1,2]. This treatment may soon in rich countries be replaced by interferon free regimes containing directly active antivirals and ribavirin [3,4] sparing the patients for the common and sometimes serious side effects of interferon. However, the cost of this improvement in economic terms will be considerable. Interferon and ribavirin will therefore probably continue to be the standard treatment of genotype 2 and 3 infection at least in low and middle-income countries also in the foreseeable future.

To save costs and avoid sideeffects we have previously explored the possibility of shortening therapy with pegIFN and ribavirin to patients with genotype 2 or 3 and have shown that among those who achieve rapid virological response (RVR) 80–90% will achieve SVR after 12–14 weeks of treatment [5–9]. However, the trials also showed that the relapse rate is approximately 5% lower if treatment is prolonged to 24 weeks even in patients with RVR.

Most studies tailoring treatment duration according to week 4 response used the Cobas Amplicor HCV monitor test (CA); with a lower detection limit of 50 IU/ml. Therefore, the EASL Clinical Practice Guidelines defined RVR as serum HCV RNA < 50 IU/ml week 4 during treatment [10]. However, nowadays sensitivity of most assays has been improved with detection limit of 10–15 IU/l [11–13]. The transcription-mediated amplification (TMA)-based assays are extremely sensitive and detect HCV RNA in serum at levels as low as 10 IU/ml [14–16]. Few data are available on how this added information provided by the new assays may guide clinical decision making for genotype 2 or 3 patients.

We aimed at evaluating the contribution of a more sensitive assay on patients treatment monitoring by determining the relapse rates in genotype 2 and 3 patients who had a negative test with CA but a positive test with TMA at week 4 of treatment. Secondary aims were to determine relapse rates according to treatment duration (14 or 24 weeks) in those with a negative CA and positive TMA and to compare positive and negative predictive values for SVR of the two tests performed at weeks 4 during treatment.

## Material and Methods

Data from one non-randomized trial (n = 122) and one randomized controlled trial (RCT) (n = 428) of short treatment to patients with genotype 2 or 3 were pooled [6,7]. In both trials patients were eligible for inclusion if they were HCV RNA positive, treatment naïve had HCV genotype 2 or 3 and raised alanine aminotransferase (ALT). Exclusion criteria have previously been published [6,7].

The Regional Ethics Committees approved the studies, and all patients gave written, informed consent.

### Treatment

Patients in both trials were treated with PEG-IFN-α-2b 1,5 μg/kg subcutaneously once weekly and ribavirin 800–1400 mg/day based on body weight (<65 kg: 800 mg/day, 65–85 kg: 1000 mg/day, 86–105 kg: 1200 mg and >105 kg: 1400 mg/day).

Those who were HCV RNA negative (<50 IU/ml) at week 4 were defined as having RVR. In the non-randomised trial all patients with RVR were treated for 14 weeks while in the RCT patients with RVR were randomized to either 14 or 24 weeks. Patients without RVR were treated for 24 weeks in both trials

Liver fibrosis was assessed using the APRI score (AST per AST_upper normal level_ per Platelets per Platelets_lower normal level_: <1.5 early stage disease and >1.5 = bridging fibrosis or cirrhosis)

### Inclusion in the current analysis

**Fig 1:**
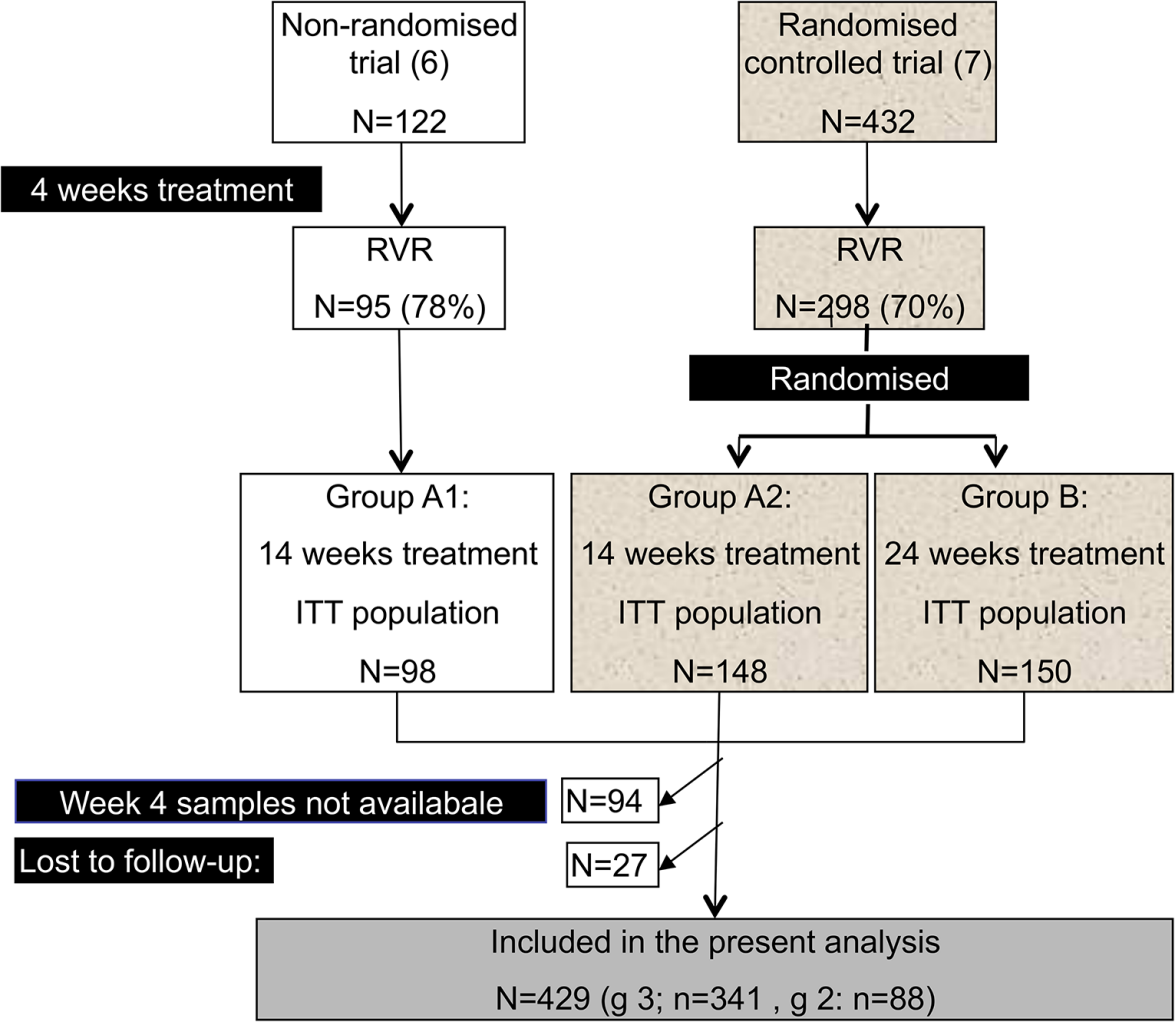
Selection of patients. Patients were recruited from two trials assessing shortened treatment to patients with genotype 2 or 3 and rapid virological response (<50 IU/ml week 4). In the present analysis those with RVR, not lost to follow-up and with available week 4 plasma samples were included.

Patients with an available frozen plasma sample drawn at week 4 and with follow-up data week 24 post-treatment were included (n = 429) (Fig 1). Among the 550 patients included in the two original trials 121 were not eligible for inclusion (in 27 follow-up data was not available and in 94 samples drawn at week 4 were not stored).

### Virology

During treatment Qualititative HCV RNA analysis was performed in both trials with a Cobas Amplicor HCV monitor test, version 2.0; lower detection limit 50 IU/ml. Analyses were performed in central labs in each of the three participating countries.

For the current analysis plasma that had been stored at -70°C was thawed and reanalyzed with VERSANT HCV RNA Qualitative Assay (HCV Qual (TMA), Siemens Medical Solutions. Puteaux France) with a limit of detection of ≤ 9.6 IU/ml (Commanor).

HCV genotypes were determined using the VERSANT HCV LiPA 2.0 (Inno-Lipa HCV, Innogenetics, Ghent, Belgium).

### Statistical analyses

Following a descriptive analysis, differences between the groups were analysed using Pearson’s chi-square test. P-values of less than 0.05 was considered significant.

We performed three logistic regression analyses with RVR, discrepant HCV RNA result week 4 (TMA pos/CA neg) and relapse after RVR (both tests negative week 4) as dependant variables and age (<40 year vs >39 years), gender (males vs females), IL 28b genotype (rs12979680 genotype CC vs CT/TT), stage of fibrosis (APRI <1.5 vs APRI >1.4) and viral load (<400 kIU/ml vs > 400 kIU/ml) as independent variables. In addition treatment duration (14 vs 24 weeks) was entered as an independent variable in the last analysis

All analyses were conducted using PASW statistic v.18.0.

## Results

From the two treatment trials 429 patients with an available week 4 serum sample was included in the present analysis, 341 (79.5%) with genotype 3 infection and 88 (20.5%) with genotype 2 (Table 1).

**Table 1:** Demographic and clinical characteristics of 429 patients with genotype 2 or 3 treated for 14 or 24 weeks according to week 4 response.

Variable	All patients (n = 429)	Patients with genotype 3 (n = 341)	Patients with genotype 2 (n = 88)
Female sex (%)	149 (39%)	118 (39)	31 (40)
Age (yr-median-range)	39 (19–61)	38 (19–60)	41 (20–61)
Weight (kg-median-range)	77 (42–171)	77 (42–171)	80 (49–160)
Fibrosis score F3-F4^1^	110 (26)	96 (28)	21 (19)
No response (%)	20 (5)	19 (5)	1 (1)
Response with relapse (%)	59 (14)	50 (15)	9 (10)
Sustained virological response (%)	350 (82)	272 (80)	78 (89)

^1^Assessed with APRI score

The included patients had a higher RVR rate the remaining (75% vs 68%, respectively) but the two groups did not differ in age, gender, genotype and stage of disease distribution (data not shown).

At week 4 serum HCV RNA was undetectable in 321/429 (74.8%) with the CA test and in 272/429 (63.4%) with TMA. A discrepant result between the two tests was seen in 73 patients, 12 CA positive/ TMA negative and 61 CA negative / TMA positive. In multivariate analysis we did not identify any predictor of having a discrepant HCV RNA result at week 4.

Independent predictors of RVR (TMA negative week 4) were genotype 2 (OR 3.7; 95 C.I:2.0–7.1), age <40 years (OR 2.8, 95% C.I. 1.7–4.6), viral load at baseline <400 kIU/ml (OR 3.8, 95% C.I. 2.2–6.7) and rs12979680 genotype CC (IL 28b responder genotype) (OR 2.7 (95% C.I. 1.7–4.4).

Only age >40 years independently predicted relapse among those with RVR (OR 5.0 (95% C.I. 1.8–13.7). Strikingly, among those younger than 40 years who achieved RVR only 3.8% relapsed compared to 16.8% of those older than 40 years.

### Genotype 3

Week 4 HCV RNA was undetectable in 73.3% (250/341) and 59.8% (204/341) with the CA test and the TMA test, respectively. Discrepant results was seen in 17.6% (60/341) samples, 53 TMA positive/CA negative and 7 TMA negative/CA positive.

**Fig 2:**
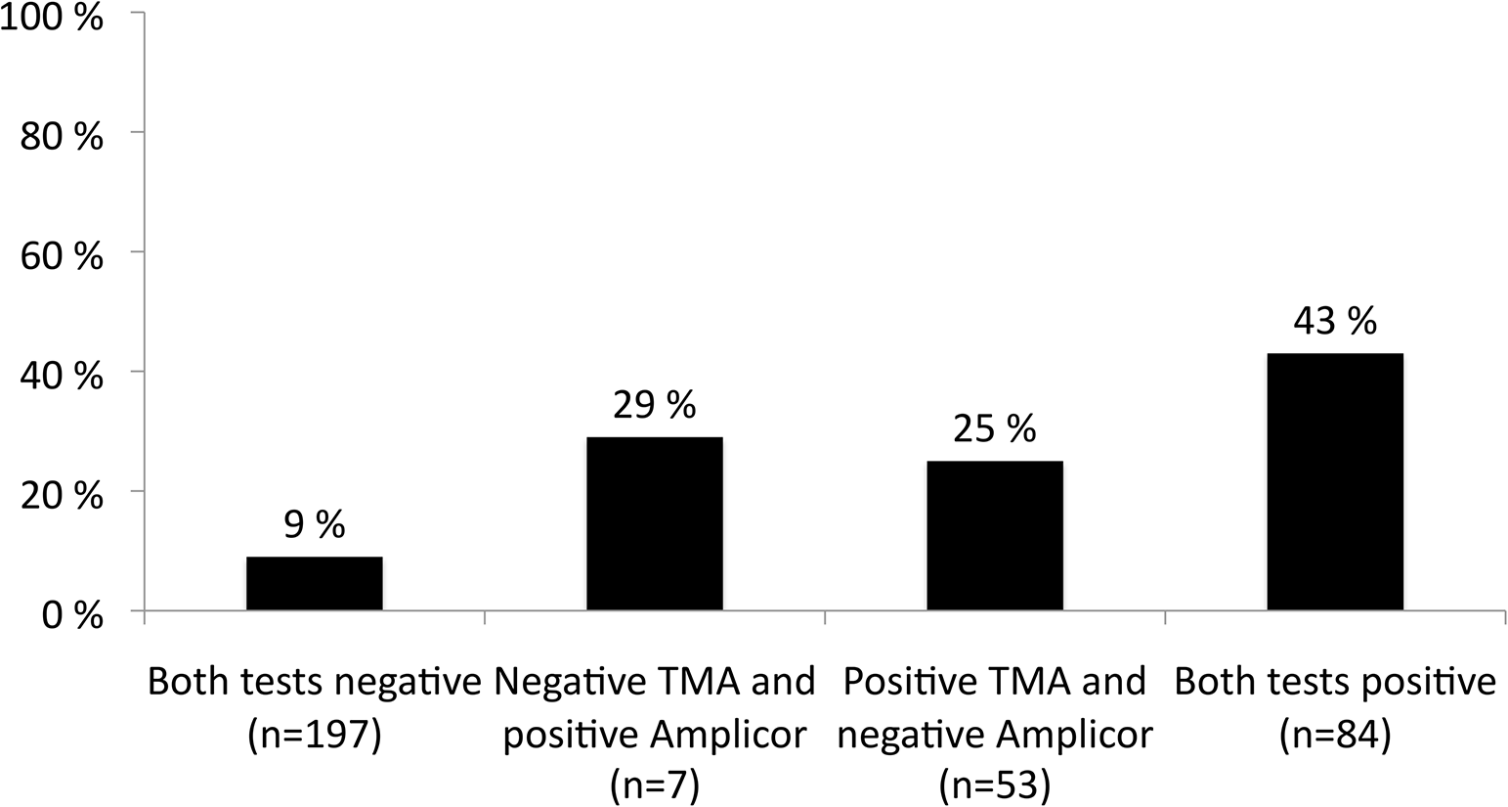
Relapse rates in patients with genotype 3 according to the combined result of the TMA and the CA tests at week 4. Patients with a negative CA test week 4 have been allocated to14 or 24 weeks while all patients with a positive CA have been treated for 24 weeks.

Relapse was observed in 9.2% (18/197) of patients with both week 4 negative tests and 24.5% (13/53) in patients with positive TMA but negative CA (p = 0.006) (Fig 2).

**Fig 3:**
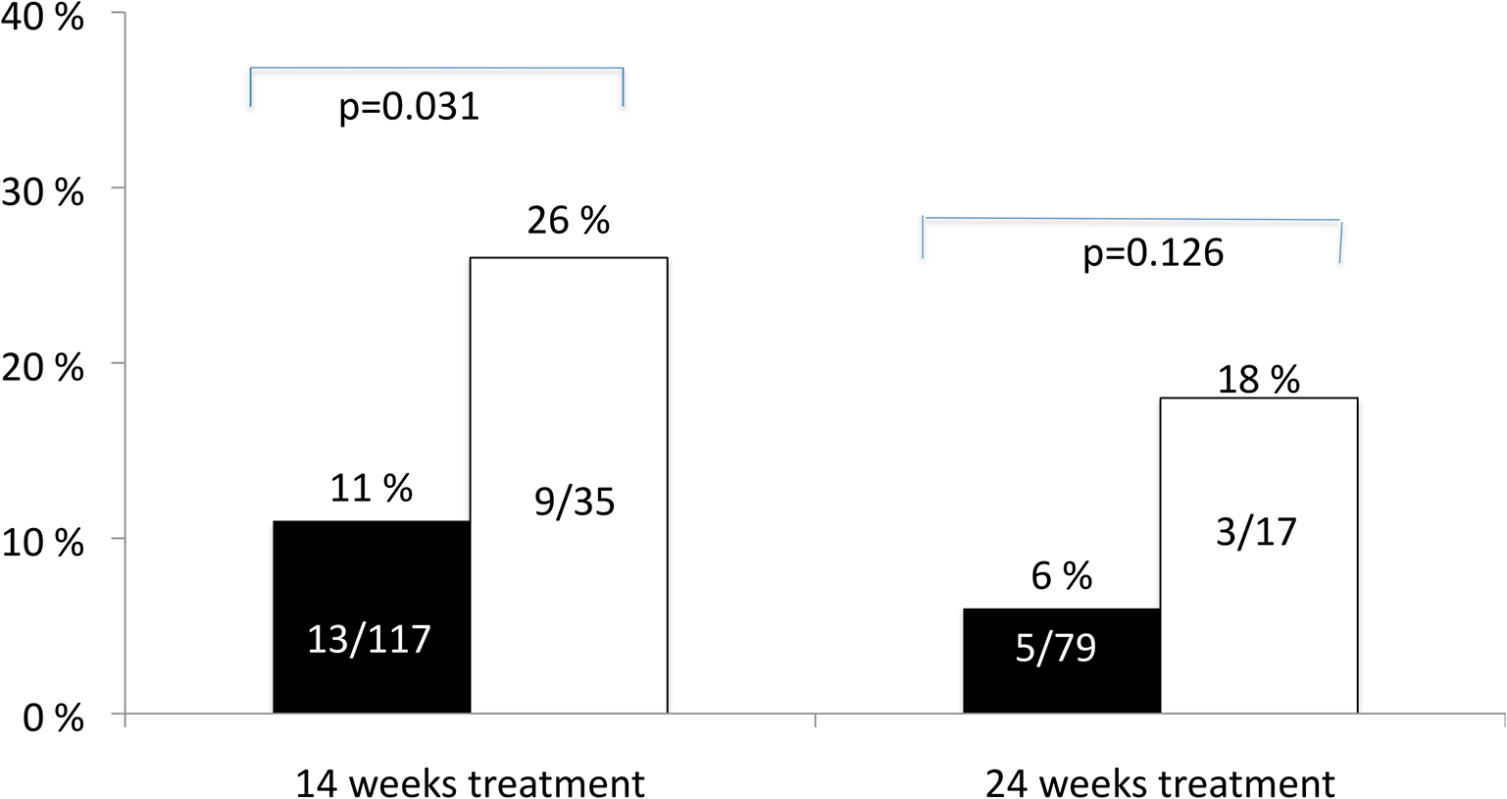
Relapse rates according to treatment duration and TMA result week 4 (black = negative and white = positive) in patients with genotype 3 with a negative CA week 4 and allocated to either 14 or 24 weeks of treatment with pegylated interferon alfa and ribavirin.

Stratifying for treatment duration we found that among patients originally classified as having RVR and allocated to 14 weeks treatment a relapse was seen in 11.1% (13/117) if TMA also was negative versus 25.7% (9/35) if TMA was positive (p = 0.031) (Fig 3). In those with RVR and allocated to the 24 weeks treatment group a relapse was observed in 6.3% (5/79) of those with a negative TMA week 4 and 17.6% (3/17) in those who were TMA positive (p = 0.126).

The week 4 PPV for SVR was 90.2% and 87.6% for negative TMA and negative CA, respectively. The sensitivity was 67.6% and 80.5% and the specificity 71.0% and 55.1% for TMA and CA test, respectively. The NPV was 35.8% for the TMA and 41.8% for the CA test.

### Genotype 2

In genotype 2 patients week 4 HCV RNA was undetectable in 80.7% (71/88) and 77.3% (68/88) with the CA and the TMA, respectively. A discrepant results was found in 14.8% (13/88) of the samples (8 CA negative / TMA positive and 5 CA positive / TMA negative).

A relapse was observed in 6.3% (4/63) of patients negative with both tests. In patients with discrepant test results a relapse was observed in 1/8 CA negative / TMA positive and 1/5 CA positive / TMA negative patients.

The week 4 PPV for SVR was 92.6% and 93% for negative TMA and negative CA, respectively. The sensitivity was 80.8% for a negative TMA and 83.3% for a negative CA. The specificity was 50% for both assays. The NPV were 25% and 29.4% for the TMA and the CA test, respectively.

## Discussion

In most trials exploring the effect of shortened treatment to patients with genotype 2 or 3 RVR was defined as having <50 IU/ml week 4 [6,8]. However, today assays that detect HCV RNA levels below 50 IU/ml are commonly used [11–13,17].

In the present reanalysis of two clinical trials we show that among genotype 3 patients, 9% of those with undetectable HCV RNA with both CA and TMA at week 4 relapsed while in patients with serum HCV RNA level below 50 IU/ml (CA testing) but detectable with the very sensitive TMA assay 25% relapsed when treated for only 14 weeks.

The relapse rate was 9% and 5% in genotype 3 and 2 patients with RVR. In most studies a larger difference in favor of genotype 2 infections has been observed[5,8,18]. We suspect the patients being younger in our study may explain the discrepancy and that genotype 3 patients in particular become less interferon sensitive with ageing. However, relatively few genotype 2 patients were included and discrepant results between the TMA and CA was not frequent enough to allow a meaningful analysis of this group. However, this and previous studies have shown that among patients with genotype 2 only 5% of those who achieve RVR (< 50 IU/ml) relapse after 12–14 weeks treatment. We therefore recommend that treatment is truncated in all patients with genotype 2 that achieve RVR even those who have detectable HCV RNA at levels lower than 50 IU/ml. A 25% risk of relapse as seen in the genotype 3 patients with positive TMA and negative CA is in our view unacceptably high. Although we do not have a sufficient sample size to inform us of the relapse rate in this group after 24 weeks treatment we do recommend that patients with genotype 3 and detectable HCV RNA at levels below 50 IU/ml do not receive truncated therapy with pegIFN and ribavirin.

The great majority of patients with a negative CA also have a negative TMA test. This explains why the PPV for SVR are quite similar for the methods (90.2% and 87.6% for TMA and CA, respectively for genotype 3. A few patients had a positive TMA and a negative CA. This result may be due to degradation of HCV RNA during thawing for TMA testing and may have lead to a slight underestimate of the PPV for SVR of the week 4 TMA test.

The ACCELERATE trial compared 16 and 24 weeks treatment to patients with genotype 2 or 3 independent of week 4 response. Sera drawn at week 4 during this trial have also been retested with a sensitive assay[18]. Using the COBAS TaqMan assay with a lower level of detection of 15 IU/ml the authors identified 11/172 patients with genotype 2 or 3 treated for 16 weeks who had HCV RNA quantifiable between 15–50 IU/ml at week 4[19]. Although the numbers are small it is interesting to note that 9 of the 11 patients relapsed. As we did not quantify HCV RNA in our retesting of the week 4 samples the studies are not directly comparable. However in agreement with our findings the authors showed that the PPV for SVR of a negative CA at week 4 was similar to the PPV of Cobas Taqman.

Our findings study is retrospective and frozen plasma samples were only available from 80% of the 550 participants in the original trials. A selection bias may therefore be present. The RVR rate in the sample was 75% in the present study compared to 72% in the original sample suggesting a bias towards more favorable response rates in the included sample. Age and genotype distribution and SVR rates were similar in the two samples.

It should be noted that ribavirin was given weightbased in these trials and not as the standard fixed 800 mg dose. We recommend that weightbased ribavirin is used when truncated planned for those with RVR.

Conclusion: Assays with high sensitivity for HCV RNA identifies genotype 3 patients at week 4 with a high risk of virological relapse. We recommend that these assays are used when truncated therapy to those with RVR is considered for patients with genotype 3.
